# QUALITY OF LIFE IN OLDER ADULTS RECEIVING IMMUNE CHECKPOINT INHIBITOR THERAPY

**DOI:** 10.1093/geroni/igz038.1145

**Published:** 2019-11-08

**Authors:** Aasha I Hoogland, Sarah L Eisel, Nathaly E Irizarry-Arroyo, Brian James, Jori Mansfield, Elizabeth A Lafranchise, Heather S Jim

**Affiliations:** 1 Cancer Center, Tampa, Florida, United States; 2 Moffitt Cancer Center, Tampa, Florida, United States; 3 Morsani College of Medicine, University of South Florida, Tampa, Florida, United States; 4 University of South Florida, Tampa, Florida, United States

## Abstract

Immune checkpoint inhibitors (ICIs) have generated significant excitement for their ability to extend survival in patients with lung, head and neck, and other cancers. In older adults with cancer, emerging research suggests that ICIs improve overall and progression-free survival, but few studies have reported on quality of life (QOL). The goal of this study was to examine changes in QOL over time in older (65+ years) vs. younger (<65 years) lung and head and neck cancer patients. Eligible participants scheduled to begin ICI for lung or head and neck cancer completed the Functional Assessment of Cancer Therapy General (FACT-G) every 2-4 weeks until disease progression. Controlling for cancer site, age group differences in QOL over time were evaluated using linear mixed models. A total of 80 lung cancer (mean age=66.5, 55% female, 55% aged 65+) and 55 head and neck cancer patients (mean age=61.5, 15% female, 45% aged 65+) provided consent. At baseline, patients with head and neck cancer reported significantly lower overall QOL, physical well-being, and emotional well-being compared to patients with lung cancer (ps<.0001). Older patients had marginally higher baseline emotional well-being than younger patients (p=.07). Across groups, there were increases in social well-being (p=.04) and a trend toward decreasing physical well-being (p=.10) over time. Patients with head and neck cancer reported lower emotional well-being over time than patients with lung cancer (p<.01). There were no age differences in QOL over time. Larger longitudinal studies are needed to better understand QOL in older patients receiving ICIs.

